# Anthropometric Measurements and Frailty in Patients with Liver Diseases

**DOI:** 10.3390/diagnostics10060433

**Published:** 2020-06-25

**Authors:** Hiroki Nishikawa, Kazunori Yoh, Hirayuki Enomoto, Naoto Ikeda, Nobuhiro Aizawa, Takashi Koriyama, Takashi Nishimura, Shuhei Nishiguchi, Hiroko Iijima

**Affiliations:** 1 Department of Internal Medicine, Division of Gastroenterology and Hepatology, Hyogo College of Medicine, Nishinomiya, Hyogo 663-8501, Japan; mm2wintwin@ybb.ne.jp (K.Y.); enomoto@hyo-med.ac.jp (H.E.); nikeneko@hyo-med.ac.jp (N.I.); nobu23hiro@yahoo.co.jp (N.A.); takashi051114@yahoo.co.jp (T.K.); tk-nishimura@hyo-med.ac.jp (T.N.); hiroko-i@hyo.med.ac.jp (H.I.); 2 Center for Clinical Research and Education, Hyogo College of Medicine, Nishinomiya, Hyogo 663-8501, Japan; 3 Kano General Hospital, Osaka, Osaka 531-0041, Japan; nishiguchi@heartfull.or.jp

**Keywords:** anthropometry measurement, frailty, chronic liver disease, calf circumference

## Abstract

There have been scarce data regarding the relationship between frailty and anthropometry measurements (AMs) in patients with chronic liver diseases (CLDs). We aimed to elucidate the influence of AMs on frailty in CLDs (median age = 66 years, 183 men and 192 women). AMs included arm circumference, triceps skinfold thickness, calf circumference (CC), waist circumference, and body mass index. Frailty assessment was done by using five phenotypes (body weight loss, exhaustion, decreased muscle strength, slow walking speed, and low physical activity). Robust (frailty point 0), prefrail (frailty point 1 or 2), and frailty (frailty point 3 or more) were observed in 63 (34.4%), 98 (53.6%), and 22 (12.0%) of males, respectively, and 63 (32.8%), 101 (52.6%), and 28 (14.6%) of females, respectively. In receiver operating characteristics (ROC) curve analyses for the presence of frailty, CC had the highest area under the ROC (AUC) both in male (AUC = 0.693, cutoff point = 33.7 cm) and female (AUC = 0.734, cutoff point = 33.4 cm) participants. In the multivariate analysis associated with frailty, for the male participants, only the presence of liver cirrhosis (*p* = 0.0433) was identified to be significant, while among the female participants, serum albumin (*p* = 0.0444) and CC (*p* = 0.0010) were identified to be significant. In conclusion, CC can be helpful for predicting frailty, especially in female CLD patients.

## 1. Introduction

Frailty is a concept globally used in geriatrics and is defined as a condition of increased vulnerability to endogenous and/or exogenous stressors associated with physiological decline, and it precedes disability in human life [1,2,3,4]. Originally, this concept was put forward in order to pick up aged individuals with a high risk of adverse health-related consequences, falls, disabilities, dependencies, and mortality [1,2,3]. Recently, frailty has been increasingly recognized in scientific medical reports, including in chronic liver diseases (CLDs) where it can be found in nearly half of patients with liver cirrhosis (LC) [5,6,7,8]. Frailty is considered to be reversible, with promising data supporting rehabilitation and lifestyle interventional programs [9,10,11]. Understanding how frailty is related to adverse outcomes requires looking at frailty as a systemic disorder as well as sarcopenia assessed by muscle mass decrease and muscle strength decline [8,12,13]. However, frailty and sarcopenia can occur with different prevalence, with variable impacts on outcomes in patients on waiting lists for liver transplantation [14].

Body composition analysis is an essential part of the assessment of nutritional status and can provide prognostically helpful insights and an opportunity to monitor the influences of nutrition-associated disease progression and nutritional interventions [15]. Anthropometric measurements (AMs) are convenient and non-invasive to evaluate body composition and thus, they are suitable for nutritional evaluation in daily clinical practice [15,16,17,18]. Arm circumference (AC) and calf circumference (CC) are primarily used to calculate muscle mass, and body mass index (BMI), triceps skinfold thickness (TSF), and waist circumference (WC) are primarily used to calculate fat mass [15]. Of those parameters, CC is recommended for its use in the revised Asian Working Group for Sarcopenia (AWGS) guidelines for the purpose of facilitating earlier identification of sarcopenic individuals [19]. In the revised AWGS guidelines, 34 cm in men and 33 cm in women are the cutoff points in CC for sarcopenia [19].

However, to the best of our knowledge, there have been scarce data regarding the relationship between frailty and AMs in patients with CLDs [20]. The aim of the study was to elucidate the influence of AMs on frailty in patients with CLDs.

## 2. Patients and Methods

### 2.1. Patients

This was a retrospective observational and cross-sectional study. A total of 375 CLD subjects with both frailty and AMs evaluable visited our hospital between July 2015 and April 2020, who were subjected to this analysis. Patients with large ascites who could suffer from a walking speed (WS) decline were not included in this study. LC was determined as reported elsewhere [21,22,23,24,25]. AMs included AC (cm), TSF (mm), CC (cm), WC (cm), and BMI (kg/m^2^). Frailty assessment was done as reported elsewhere [25]. Briefly, using 5 phenotypes proposed by Fried et al. (i.e., body weight (BW) loss without intention, exhaustion, muscle strength decline (grip strength (GS): <26 kg in men and <18 kg in women), slow walking speed (WS, <1.0 m/s), and low physical activity (being unable to do little exercise)), patients with 3 or more phenotypes were defined as frail, while patients with 1 or 2 phenotypes were defined as prefrail and those with no phenotype as robust [25,26,27]. GS and WS were calculated as reported elsewhere [25,28]. In our hospital, AMs have been done by an expert nutrition therapist after full explanation of the need and implication of AMs to patients. Measurement of AC, CC, TSF, and WC were based on Japanese anthropometric reference data 2001.

We examined the impact of AMs on frailty in male and female CLD patients in a retrospective manner. The institutional review board in Hyogo college of medicine hospital approved the research protocol (approval no. 3469, date of approval: 27 March 2020) and the 1975 Declaration of Helsinki was rigorously adhered to secure the rights of the patients. An opt out method was employed for the purpose of obtaining informed consent from the patients.

### 2.2. Statistical Considerations

All statistical analyses were done using the JMP 14 software (SAS Institute Inc., Cary, NC, USA). In the analysis of numerical variables, Mann-Whitney *U*-test or Student’s *t*-test, Kruskal-Wallis test, or analysis of variance (ANOVA) was employed to assess group characteristics when appropriate. In the analysis of categorical variables, percentages were compared using the chi-squared test or Fisher’s exact test, as applicable. Receiver operating characteristic curve (ROC) analysis and area under the ROC (AUC) data were shown along with the corresponding optimal cutoff point, sensitivity, and specificity. Quantitative data were expressed as medians with interquartile range (IQR). Significant parameters in the univariate analysis were subject to the multivariate logistic regression analysis to select candidate parameters. The statistical significance level was set at *p* < 0.05.

## 3. Results

### 3.1. Baseline Features

Baseline features of the study cohort (*n* = 183 in male (median (IQR) age = 66 (53, 72) years) and *n* = 192 in female (median (IQR) age = 66 (55, 72.8) years); *p* = 0.6237) were presented in Table 1. LC was identified at baseline in 72 cases (39.3%) in male participants and 58 cases (30.2%) in female participants (*p* = 0.0660). Hepatitis C virus accounted for 39.3% in male participants (72/183) and 55.2% (106/192) in female participants. In terms of albumin-bilirubin (ALBI) grade, ALBI grade 1 was in the majority, both in male (125/183, 68.3%) and female (155/192, 80.7%) participants. In male participants, frailty patients had a significantly higher age (*p* = 0.0126), lower serum albumin levels (*p* < 0.0001), and a higher proportion of LC (*p* = 0.0003) than prefrail or robust patients. Similarly, in female participants, patients had a significantly higher age (*p* = 0.0146), lower serum albumin levels (*p* = 0.0010), and a higher proportion of LC (*p* = 0.0001) than prefrail or robust patients.

The median (IQR) WS in male and female participants were 1.28 (1.08, 1.44) m/s and 1.32 (1.16, 1.47) (*p* = 0.4618), respectively. Twenty-eight male patients (15.3%) and 27 female patients (14.1%) had a WS decrease (i.e., <1.0 m/s). The median (IQR) GS in male and female participants were 33.3 (28.0, 39.0) kg and 21.0 (17.7, 24.5) kg. Thirty-five male patients (19.1%) and 50 female patients (26.0%) had a GS decrease (i.e., <26 kg in male and <18 kg in female). Eighty-two male patients (44.8%) and 92 female patients (47.9%) reported exhaustion. Fourteen male patients (7.7%) and 11 female patients (5.7%) reported BW loss. Fifty-one male patients (27.9%) and 44 female patients (22.9%) reported low physical activity. The frailty point ranged from 0 to 4 (median point = 1) in male and 0 to 5 (median point = 1) in female participants. Robust (frailty point 0), prefrail (frailty point 1 or 2), and frailty (frailty point 3 or more) were observed in 63 (34.4%), 98 (53.6%), and 22 (12.0%) in male participants, respectively, and 63 (32.8%), 101 (52.6%), and 28 (14.6%) in female participants, respectively.

In terms of AMs, the median (IQR) AC (cm), TSF (cm), WC (cm), CC (cm), and BMI (kg/m^2^) in male vs. female were: 28 (26, 30) cm vs. 28 (25, 30.8) cm in AC (*p* = 0.1688), 12 (9, 16) mm vs. 20 (15, 27) mm in TSF (*p* < 0.0001), 36.9 (34.4, 39.2) cm vs. 34.8 (32.7, 37.4) cm in CC (*p* < 0.0001), 89.5 (83.5, 97.0) cm vs. 85.8 (80, 92.5) cm in WC (*p* < 0.0001), and 23.2 (21.4, 25.9) kg/m^2^ vs. 22.3 (20.1, 25.5) kg/m^2^ (*p* = 0.0136) in BMI.

### 3.2. AMs According to Frailty Status in Males

**Figure 1:**
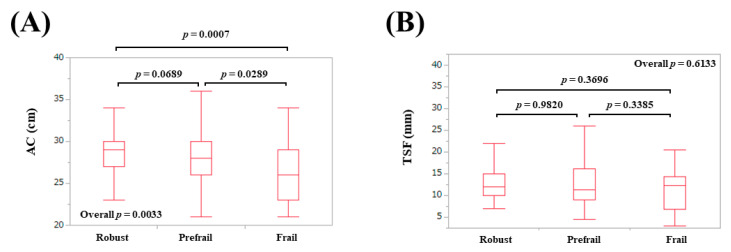
Anthropometry measurements according to frailty status in male participants. (**A**) Arm circumference; (**B**) Triceps skinfold thickness.

**Figure 2:**
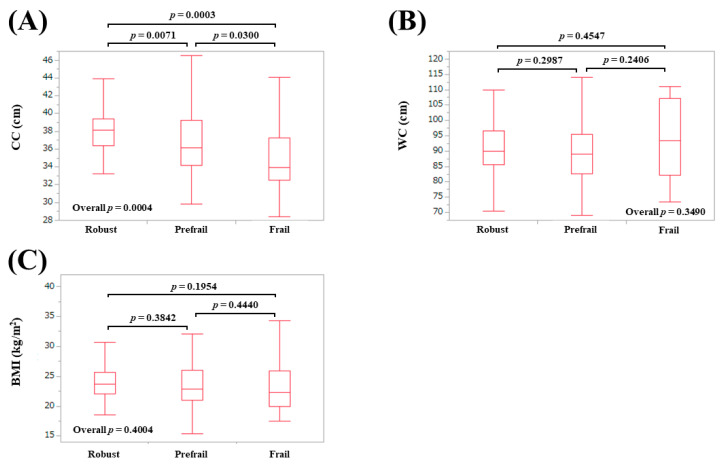
Anthropometry measurements according to frailty status in male participants. (**A**) Calf circumference; (**B**) Waist circumference; (**C**) Body mass index.

The median (IQR) AC in patients defined as robust, prefrail, and frail were: 29 (27, 30) cm in robust, 28 (26, 30) cm in prefrail, and 26 (23, 29) cm in frail (overall *p* = 0.0033) (Figure 1A). The median (IQR) TSF in patients defined as robust, prefrail, and frail were: 12 (10, 15) mm in robust, 11.25 (9, 16.1) mm in prefrail, and 12.3 (6.9, 14.3) mm in frail (overall *p* = 0.6133) (Figure 1B). The median (IQR) CC in patients defined as robust, prefrail, and frail were: 38.1 (36.4, 39.4) cm in robust, 36.2 (34.2, 39.25) cm in prefrail, and 34.0 (32.5, 37.3) cm in frail (overall *p* = 0.0004) (Figure 2A). The median (IQR) WC in patients defined as robust, prefrail, and frail were: 90 (85.5, 96.5) cm in robust, 89 (82.7, 95.5) cm in prefrail, and 93.4 (82.3, 107.3) cm in frail (overall *p* = 0.3490) (Figure 2B). The median (IQR) BMI in patients defined as robust, prefrail, and frail were: 23.7 (22, 25.7) kg/m^2^ in robust, 22.9 (21.0, 26) kg/m^2^ in prefrail, and 22.3 (20.0, 26.0) kg/m^2^ in frail (overall *p* = 0.4004) (Figure 2C).

### 3.3. AMs According to Frailty Status in Females

**Figure 3:**
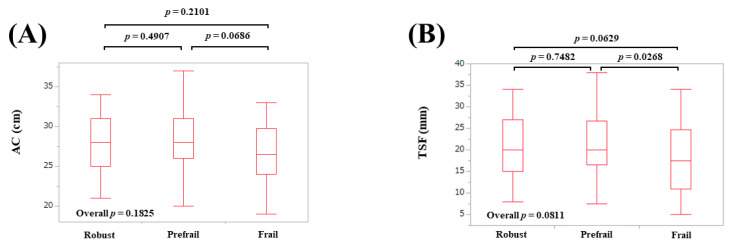
Anthropometry measurements according to frailty status in female participants. (**A**) Arm circumference; (**B**) Triceps skinfold thickness.

**Figure 4:**
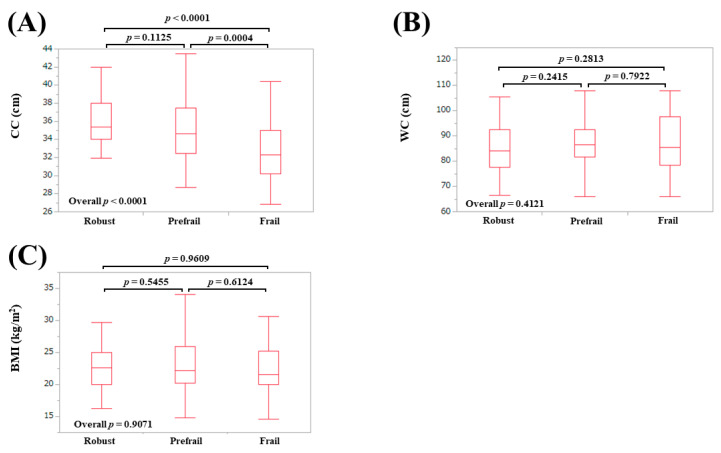
Anthropometry measurements according to frailty status in female participants. (**A**) Calf circumference; (**B**) Waist circumference; (**C**) Body mass index.

The median (IQR) AC in patients defined as robust, prefrail, and frail were: 28 (25, 31) cm in robust, 28 (26, 31) cm in prefrail, and 26.5 (24, 29.75) cm in frail (overall *p* = 0.1825) (Figure 3A). The median (IQR) TSF in patients defined as robust, prefrail, and frail were: 20 (15, 27) mm in robust, 20 (16.5, 26.8) mm in prefrail, and 17.5 (11, 24.8) mm in frail (overall *p* = 0.0811) (Figure 3B). The median (IQR) CC in patients defined as robust, prefrail, and frail were: 35.4 (34, 38) cm in robust, 34.6 (32.5, 37.5) cm in prefrail, and 32.3 (30.2, 35) cm in frail (overall *p* < 0.0001) (Figure 4A). The median (IQR) WC in patients defined as robust, prefrail, and frail were: 84 (77.5, 92.4) cm in robust, 86.5 (81.8, 92.5) cm in prefrail, and 85.5 (78.6, 97.5) cm in frail (overall *p* = 0.4121) (Figure 4B). The median (IQR) BMI in patients defined as robust, prefrail, and frail were: 22.6 (20, 25) kg/m^2^ in robust, 22.2 (20.3, 26.0) kg/m^2^ in prefrail, and 21.6 (20.0, 25.3) kg/m^2^ in frail (overall *p* = 0.9071) (Figure 4C).

### 3.4. AMs According to Frailty Phenotypes in Males

For the male participants, the values of AC (*p* = 0.010) and CC (*p* = 0.0339) in patients with a decrease in WS were significantly lower than those in each counterpart (Table 2). The values of AC (*p* < 0.0001), CC (*p* < 0.0001), and BMI (*p* = 0.0037) in patients with a decrease in GS were significantly lower than those in each counterpart (Table 2). The values of AC (*p* = 0.0278) and CC (*p* = 0.0225) in patients with physical activity decrease were significantly lower than those in each counterpart (Table 2). No significant differences of 5 AMs between the two groups were found in terms of fatigue and BW loss.

### 3.5. AMs According to Frailty Phenotypes in Females

In female participants, the values of TSF (*p* = 0.0247) and CC (*p* = 0.0002) in patients with a decrease in WS were significantly lower than those in each counterpart (Table 3). The value of CC (*p* = 0.0002) in patients with GS decrease was significantly lower than that in the counterpart (Table 3). No significant differences of 5 AMs between the two groups were found in terms of fatigue, BW loss, and physical activity.

### 3.6. ROC Analysis for the Presence of Frailty in Males

ROC analysis for the presence of frailty in male participants revealed that CC had the highest AUC (AUC = 0.693), followed by AC (AUC = 0.676) among 5 AMs (Table 4). Corresponding cutoff value, sensitivity, and specificity are shown in Table 4.

### 3.7. ROC Analysis for the Presence of Frailty in Females

ROC analysis for the presence of frailty in female participants revealed that CC had the highest AUC (AUC = 0.734), followed by TSF (AUC = 0.62) among 5 AMs (Table 4). Corresponding cutoff value, sensitivity, and specificity are shown in Table 4.

### 3.8. Univariate and Multivariate Analyses of Factors Linked to Frailty in Males

In male participants, in the univariate analysis, age (*p* = 0.0126), presence of LC (*p* = 0.0003), serum albumin level (*p* < 0.0001), AC (*p* = 0.0038), and CC (*p* = 0.0056) were observed to be significant factors associated with the presence of frailty (Table 5). In the multivariate analysis for the five factors, only presence of LC (*p* = 0.0433) was identified to be a significant factor linked to the presence of frailty (Table 5). Corresponding odds ratio (OR) and confidence interval (CI) are shown in Table 5.

### 3.9. Univariate and Multivariate Analyses of Factors Linked to Frailty in Females

In female participants, in the univariate analysis, age (*p* = 0.0188), presence of LC (*p* = 0.0003), serum albumin level (*p* = 0.0010), AC (*p* = 0.0491), TSF (*p* = 0.0263), and CC (*p* < 0.0001) were observed to be significant factors associated with the presence of frailty (Table 5). In the multivariate analysis for the six factors, serum albumin (*p* = 0.0444) and CC (*p* = 0.0010) were identified to be significant factors linked to the presence of frailty (Table 5). Corresponding OR and CI are shown in Table 5.

## 4. Discussion

Japan is aging at an unprecedented speed and it will continue to age in the future. The same can apply to Japanese CLD patients [29,30]. As mentioned earlier, AMs are convenient and non-invasive to evaluate body composition [15,16,17,18]. How to use convenient markers in practice is of importance clinically. However, scarce data have provided a description of AMs and frailty in patients with CLDs [20]. In our data, AC and CC were well stratified according to the frailty status in male and CC in female participants. In ROC analyses, CC had the highest AUC for frailty both in male (AUC = 0.693) and in female (AUC = 0.734) participants among five AMs. In the multivariate analysis, CC was an independent factor for frailty in female participants. Considering this, our results denoted that CC can be a useful AM for frailty in CLDs. Regarding sarcopenia surveillance, the revised AWGS guidelines propose separate algorithms for community vs. hospital settings [19]. In community settings, assessment of muscle mass is not required for the diagnosis of sarcopenia in the revised AWGS guidelines. In both community and hospital settings, first screening for possible sarcopenia using CC or a questionnaire is recommended. CC appears to be helpful for the assessment of both sarcopenia and frailty. In addition, one should note that CC value decreases, even in the prefrail stage. On the other hand, in the multivariate analyses, the presence of LC in male participants and serum albumin levels in female participants were significant factors for frailty. Disease specific frailty in CLDs should be taken into account. Several reports have suggested that hepatic decompensation is associated with a significantly elevated risk of frailty and frailty is linked to a significantly elevated number and duration of hospital admissions for LC-related complications [8,31,32,33]. In our male non-LC patients (*n* = 111), frailty was found in 5 patients (4.5%) and prefrailty was found in 57 patients (51.4%), while in our female non-LC patients (*n* = 134), frailty was found in 11 patients (8.2%) and prefrailty was found in 70 patients (52.2%). One should also keep in mind that non-LC status does not deny the possibility of frailty.

The cutoff values of CC for sarcopenia in the revised AWGS guidelines are 34 cm in males and 33 cm in females, while in our ROC analysis for frailty, the optimal cutoff values of CC were 33.7 cm in males and 33.4 cm in females, which are almost identical to AWGS data [19,34]. Sarcopenia indicates physical frailty and sarcopenia forms the basis of frailty [2,35]. Sarcopenia itself causes lower limb muscle strength, falls, slow WS, and thus, decrease in physical activity can be induced. These all contribute to the development of frailty. Coincidence of cutoff values of CC between AWGS data and our data is not so surprising. Sarcopenia and frailty are unique, inter-related, and multi-dimensional issues in CLDs [6]. Rolland et al. reported that female presenting with a CC <31 cm were three times more likely to have difficulties in moving [36]. In our cohort, there were 21 female patients (10.9%) with a CC <31 cm. Of these, robustness was found in one patient, prefrailty in 11, and frailty in 9, which may support the usefulness of CC as a screening tool for frailty in female CLD patients. Fatigue and BW loss (phenotypes for frailty) did not correlate with AMs, both in male and female participants, in our data. As expected, AMs can correlate with physical function.

BMI and WC were not significant factors linked to frailty, both in male and female participants, in our analysis. A close correlation between higher abdominal obesity and frailty in males has been reported [37]. Another study reported that being overweight was significantly associated with prefrailty and obesity was associated with prefrailty and frailty in females [38]. The reasons for these discrepancies between their data and our data are unclear and further studies regarding the impact of obesity on frailty in CLDs is necessary. AC means the sum of arm muscle circumference and TSF [15]. Thus, both muscle mass and fat mass can affect AC value. This may be linked to our findings that AC was not a significant factor in the multivariate analysis, both for male and female participants, although it was significant in the univariate analysis, both for male and female participants.

Several limitations associated with the study must be mentioned. Firstly, this observational study had a retrospective and cross-sectional nature, with subjects from a single hospital. Secondly, our data included population data from CLD patients in Japan; thus, additional studies on subjects from other parts of the world are needed to confirm and expand or adapt our results for each population. Thirdly, patients with large ascites who could suffer from a WS decline were not included in this study, therefore this possibly creates bias. Finally, due to the cross-sectional nature of our study, the causal relationship between AMs and frailty is unclear. Interpretation with caution to our study data is required. Our study results nevertheless implied that AMs and frailty in CLDs are closely correlated, especially in CC. In conclusion, CC can be helpful for predicting frailty, especially in female CLD patients.

## Figures and Tables

**Table 1:** Baseline characteristics (*n* = 375).

Variables	Male (*n* = 183)	Female (*n* = 192)	*p* Value
Age (years)	66 (53, 72)	66 (55, 72.8)	0.6237
BMI (kg/m^2^)	23.2 (21.4, 25.9)	22.3 (20.1, 25.5)	0.0136
Etiologies, HBV/HCV/others	45/72/66	22/106/64	0.0008
Presence of LC, yes/no	72/111	58/134	0.0660
Total bilirubin (mg/dL)	0.9 (0.6, 1.2)	0.8 (0.6, 1.0)	0.7926
Serum albumin (g/dL)	4.2 (3.9, 4.5)	4.3 (4.0, 4.5)	0.1711
ALBI score	−2.86 (−3.1, −2.48)	−2.91 (−3.15, −2.68)	0.0920
ALBI grade, 1/2/3	125/54/4	155/33/4	0.0177
Prothrombin time (%)	90.5 (77.9, 100.4)	93.1 (83.8, 100.1)	0.0424
Platelet count (× 10^4^/mm^3^)	16.5 (11.2, 21.4)	18.1 (13.7, 22.9)	0.0130
AST (IU/L)	26 (20, 36)	24 (19, 31.8)	0.2402
ALT (IU/L)	24 (16, 39)	17 (13, 26)	0.0456
GS (kg)	33.3 (28.0, 39.0)	21.0 (17.7, 24.5)	<0.0001
WS (m/s)	1.28 (1.08, 1.44)	1.32 (1.16, 1.47)	0.4618
Presence of frailty, yes/no	22/161	28/164	0.5439
Arm circumference (cm)	28 (26, 30)	28 (25, 30.8)	0.1688
Triceps skin fold thickness (mm)	12 (9, 16)	20 (15, 27)	<0.0001
Calf circumference (cm)	36.9 (34.4, 39.2)	34.8 (32.7, 37.4)	<0.0001
Waist circumference (cm)	89.5 (83.5, 97.0)	85.8 (80, 92.5)	<0.0001

Data are expressed as a number or median value (interquartile range). BMI, body mass index; HBV, hepatitis B virus; HCV, hepatitis C virus; LC, liver cirrhosis; ALBI, albumin-bilirubin; AST, aspartate aminotransferase; ALT, alanine aminotransferase; GS, grip strength; WS, walking speed.

**Table 2:** Anthropometric measurements according to the frailty phenotypes in male participants.

	WS Decrease	WS Non-Decrease	*p* Value
AC	27 (23.5, 29)	28 (27, 30)	0.0100
TSF	1.25 (0.91, 1.58)	1.15 (0.9, 1.6)	0.6785
CC	34.7 (32.9, 38.6)	37.1 (34.7, 39.4)	0.0339
WC	89.9 (81.6, 102.2)	89.5 (84, 96.5)	0.5781
BMI	22.2 (19.9, 25.8)	23.5 (21.6, 25.9)	0.2859
	**GS Decrease**	**GS Non-Decrease**	***p* Value**
AC	26 (25, 28)	29 (27, 30.8)	<0.0001
TSF	1.15 (0.85, 1.5)	1.2 (0.91, 1.6)	0.3758
CC	34.2 (32.9, 36.1)	37.7 (35.5, 39.6)	<0.0001
WC	89 (80.6, 96)	90 (84.2, 97.4)	0.2324
BMI	21.8 (19.8, 24.6)	23.7 (21.7, 26)	0.0037
	**Fatigue, Yes**	**Fatigue, No**	***p* Value**
AC	28 (26, 30)	28 (27, 30)	0.1255
TSF	1.2 (0.9, 1.6)	1.2 (0.93, 1.6)	0.7026
CC	36.1 (33.8, 39.2)	37.6 (34.7, 39.3)	0.1705
WC	89.1 (82.7, 97.8)	89.8 (84.3, 96.3)	0.5509
BMI	22.8 (20.6, 25.9)	23.6 (21.8, 25.7)	0.3683
	**BW Loss, Yes**	**BW Loss, No**	***p* Value**
AC	27 (23, 29.8)	29 (27, 30)	0.1929
TSF	0.8 (0.65, 1.38)	1.2 (0.95, 1.6)	0.2430
CC	34.5 (32.1, 40.9)	37.4 (35.5, 39.4)	0.1738
WC	89 (82.8, 102.9)	89.5 (84, 96.5)	0.8760
BMI	21.0 (19.2, 26.3)	23.5 (21.7, 25.7)	0.1974
	**PA Decline, Yes**	**PA Decline, No**	***p* Value**
AC	28 (26, 30)	28.5 (27, 30)	0.0278
TSF	1.1 (0.89, 1.6)	1.2 (0.95, 1.6)	0.2382
CC	36.0 (33.8, 39.1)	37.4 (35, 39.4)	0.0224
WC	92 (82.1, 100.9)	89 (84, 96.4)	0.5377
BMI	23.5 (21.7, 25.8)	23.5 (21.6, 25.9)	0.3143

Data are shown as median value (interquartile range). AC, arm circumference; TSF, triceps skinfold thickness; CC, calf circumference; WC, waist circumference; BMI, body mass index; WS, walking speed; GS, grip strength; BW, body weight; PA, physical activity.

**Table 3:** Anthropometric measurements according to the frailty phenotypes in female participants.

	WS Decrease	WS Non-Decrease	*p* Value
AC	27 (25, 29)	28 (25, 31)	0.0704
TSF	1.7 (1.2, 2.4)	2.0 (1.6, 2.7)	0.0247
CC	32.4 (30.8, 34.4)	35.1 (33.2, 37.5)	0.0002
WC	85 (80, 92.5)	85.9 (80.2, 92.7)	0.8582
BMI	22.4 (20.1, 23.8)	22.2 (20.1, 25.9)	0.3477
	**GS Decrease**	**GS Non-Decrease**	***p* Value**
AC	27 (25, 29.3)	28 (25, 31)	0.0536
TSF	2.0 (1.35, 2.6)	2.0 (1.6, 2.7)	0.2259
CC	33.3 (31.1, 35.8)	35.1 (33.5, 37.8)	0.0002
WC	86 (79.8, 92.1)	85.5 (80. 93.5)	0.9234
BMI	21.7 (20.1, 24.7)	22.7 (20, 25.8)	0.6682
	**Fatigue, Yes**	**Fatigue, No**	***p* Value**
AC	27 (25, 31)	28 (26, 30)	0.5815
TSF	1.95 (1.4, 2.8)	2.1 (1.7, 2.6)	0.2723
CC	34.5 (31.8, 37.4)	35.0 (33.5, 37.4)	0.0718
WC	86 (78.7, 94)	85.3 (81.1, 92.3)	0.7912
BMI	21.7 (19.8, 26.1)	22.7 (20.4, 24.8)	0.7081
	**BW Loss, Yes**	**BW Loss, No**	***p* Value**
AC	27 (24, 29)	28 (25, 31)	0.2554
TSF	1.8 (1.2, 2.15)	2.0 (1.5, 2.7)	0.0920
CC	33.1 (31.4, 35.1)	34.9 (32.9, 37.5)	0.0637
WC	89.5 (78, 95)	85.7 (80, 92.5)	0.6643
BMI	23 (20.5, 25.8)	22.2 (20, 25.5)	0.9613
	**PA Decline, Yes**	**PA Decline, No**	***p* Value**
AC	27.5 (26, 31)	28 (25, 30)	0.4672
TSF	2.15 (1.53, 2.89)	2.0 (1.5, 2.6)	0.4689
CC	34.6 (32.0, 35.9)	34.9 (33.0, 37.9)	0.1211
WC	88 (83.1, 93.3)	85 (78.5, 92.4)	0.1304
BMI	22.9 (20.2, 25.6)	22.2 (20, 25.4)	0.5102

Data are shown as median value (interquartile range). AC, arm circumference; TSF, triceps skinfold thickness; CC, calf circumference; WC, waist circumference; BMI, body mass index; WS, walking speed; GS, grip strength; BW, body weight; PA, physical activity.

**Table 4:** ROC analysis for the presence of frailty in male and female participants.

Male	AUC	Cutoff	Sensitivity	Specificity
AC	0.676	26.4 cm	0.546	0.764
TSF	0.565	7 mm	0.273	0.913
CC	0.693	33.7 cm	0.500	0.663
WC	0.570	95.2 cm	0.500	0.733
BMI	0.575	21.4 kg/m^2^	0.500	0.776
**Female**	**AUC**	**Cutoff**	**Sensitivity**	**Specificity**
AC	0.599	27.6 cm	0.679	0.512
TSF	0.620	12 mm	0.357	0.884
CC	0.734	33.4 cm	0.714	0.750
WC	0.528	98.4 cm	0.250	0.908
BMI	0.525	21.6 kg/m^2^	0.536	0.579

AUC, area under the receiver operating characteristics curve; AC, arm circumference; TSF, triceps skinfold thickness; CC, calf circumference; WC, waist circumference; BMI, body mass index.

**Table 5:** Multivariate analyses of factors linked to frailty in male and female participants.

Male	Univariate	Multivariate Analysis		
	*p* Value	OR	95% CI	*p* Value
Age	0.0126	0.989	0.943–1.037	0.6389
BMI	0.3000	-	-	-
Presence of LC	0.0003	0.281	0.082–0.962	0.0433
Total bilirubin	0.9262	-	-	-
Serum albumin	<0.0001	2.082	0.781–5.553	0.1428
Prothrombin time	0.3345	-	-	-
Platelet count	0.0669	-	-	-
AST	0.6275	-	-	-
ALT	0.9838	-	-	-
Arm circumference	0.0038	1.022	0.766–1.362	0.8835
Triceps skin fold thickness	0.3222	-	-	-
Calf circumference	0.0056	1.129	0.867–1.470	0.3675
Waist circumference	0.2073	-	-	-
**Female**	**Univariate**	**Multivariate Analysis**		
	***p*** **Value**	**OR**	**95% CI**	***p*** **Value**
Age	0.0188	0.987	0.947–1.030	0.5514
BMI	0.7283	-	-	-
Presence of LC	0.0003	0.462	0.157–1.361	0.1615
Total bilirubin	0.8808	-	-	-
Serum albumin	0.0010	2.617	0.998–6.864	0.0444
Prothrombin time	0.4101	-	-	-
Platelet count	0.3379	-	-	-
AST	0.1093	-	-	-
ALT	0.5371	-	-	-
Arm circumference	0.0491	1.186	0.869–1.620	0.2793
Triceps skin fold thickness	0.0263	1.004	0.883–1.141	0.9539
Calf circumference	<0.0001	1.524	1.172–1.983	0.0010
Waist circumference	0.528	-	-	-

BMI, body mass index; LC, liver cirrhosis; AST, aspartate aminotransferase; ALT, alanine aminotransferase; OR, odds ratio; CI, confidence interval.

## Abbreviations

CLD	chronic liver disease
LC	liver cirrhosis
AM	anthropometric measurement
AC	arm circumference
CC	calf circumference
BMI	body mass index
TSF	triceps skinfold thickness
WC	waist circumference
AWGS	Asian Working Group for Sarcopenia
BW	body weight
GS	grip strength
WS	walking speed
ROC	receiver operating characteristic curve
AUC	area under the receiver operating characteristic curve
IQR	interquartile range
ALBI	albumin-bilirubin
OR	odds ratio
CI	confidence interval
